# Characterization of Leaf Blade- and Leaf Sheath-Associated Bacterial Communities and Assessment of Their Responses to Environmental Changes in CO2, Temperature, and Nitrogen Levels under Field Conditions

**DOI:** 10.1264/jsme2.ME14117

**Published:** 2015-02-04

**Authors:** Seishi Ikeda, Takeshi Tokida, Hirofumi Nakamura, Hidemitsu Sakai, Yasuhiro Usui, Takashi Okubo, Kanako Tago, Kentaro Hayashi, Yasuyo Sekiyama, Hiroshi Ono, Satoru Tomita, Masahito Hayatsu, Toshihiro Hasegawa, Kiwamu Minamisawa

**Affiliations:** 1 Memuro Research Station, Hokkaido Agricultural Research Center, National Agriculture and Food Research OrganizationShinsei, Memuro-cho, Kasai-gun, Hokkaido 082–0081Japan; 2 National Institute for Agro-Environmental Sciences3–1–3 Kannondai, Tsukuba, Ibaraki 305–8604Japan; 3 Taiyo-Keiki Co., Ltd.1–12–3 Nakajujo, Kita, Tokyo, 114–0032Japan; 4 Graduate School of Life Sciences, Tohoku University2–1–1 Katahira, Aoba-ku, Sendai, Miyagi 980–8577Japan; 5 National Food Research Institute, National Agriculture and Food Research Organization2–1–12 Kannondai, Tsukuba, Ibaraki 305–8642Japan

**Keywords:** FACE, nitrogen, *Planctomycetes*, rice phyllosphere, temperature

## Abstract

Rice shoot-associated bacterial communities at the panicle initiation stage were characterized and their responses to elevated surface water-soil temperature (ET), low nitrogen (LN), and free-air CO_2_ enrichment (FACE) were assessed by clone library analyses of the 16S rRNA gene. Principal coordinate analyses combining all sequence data for leaf blade- and leaf sheath-associated bacteria revealed that each bacterial community had a distinct structure, as supported by PC1 (61.5%), that was mainly attributed to the high abundance of *Planctomycetes* in leaf sheaths. Our results also indicated that the community structures of leaf blade-associated bacteria were more sensitive than those of leaf sheath-associated bacteria to the environmental factors examined. Among these environmental factors, LN strongly affected the community structures of leaf blade-associated bacteria by increasing the relative abundance of *Bacilli*. The most significant effect of FACE was also observed on leaf blade-associated bacteria under the LN condition, which was explained by decreases and increases in *Agrobacterium* and *Pantoea*, respectively. The community structures of leaf blade-associated bacteria under the combination of FACE and ET were more similar to those of the control than to those under ET or FACE. Thus, the combined effects of environmental factors need to be considered in order to realistically assess the effects of environmental changes on microbial community structures.

Increases in atmospheric CO_2_ levels and the associated changes in the climate have had strong impacts on rice production and the carbon cycle by changing biological processes such as photosynthesis and the decomposition of soil organic matter (59). A previous study reported that elevated atmospheric CO_2_ levels stimulated photosynthesis in rice (27). Subsequent studies demonstrated that elevated CO_2_ levels increased rice biomass production of both above- and belowground tissues as well as grain yield (4, 68). Free-air CO_2_ enrichment (FACE) experiments in Japan have evaluated changes in crop productivity and the ecosystem of rice paddy fields. FACE facilitates studies on the effects of elevated CO_2_ levels on such an ecosystem under an open field environment by minimizing disturbances derived from the experimental settings and devices (1). FACE studies previously revealed that the quantity and quality of root exudates of several plant species are influenced by atmospheric CO_2_ concentrations (46, 47). Therefore, rhizosphere microorganisms are also likely to be influenced by elevated CO_2_ levels due to CO_2_ -induced changes in root exudates (47). Elevated atmospheric CO_2_ concentrations have been shown to increase the root exudation of labile carbon, thereby stimulating microbial growth (39, 46). The effects of FACE on the community structure of rice root-associated bacteria have also been reported (44).

In addition to elevated CO_2_ levels, the influences of other environmental factors on microbial community structures in rice plants also need to be evaluated in order to assess their impacts on global warming. Temperature is one of the most important environmental factors under the global warming as it is expected to increase (53). The impact of surface water-soil temperature changes on the bacterial community in rice roots has recently been reported (44). The management of nitrogen fertilization has also been identified as a critical environmental factor affecting both rice plant growth and its associated microbes with global warming (29, 39, 52). In addition to the issue of global warming, several studies revealed that the nitrogen status of plants is an important environmental factor influencing bacteria associated with both the above- and belowground tissues of plants under field conditions (23–26, 50). Previous studies reported that elevated CO_2_ levels lowered nitrogen concentrations in rice leaves (1, 36) and accelerated leaf senescence (32). These findings emphasize the importance of investigating the impact of global warming on rice plants under low nitrogen conditions.

If the current simulation for environmental changes under the expected global warming is correct (http://www.ipcc.ch/), these environmental changes will affect the diversity and functionality of microbial communities not only in underground, as described above, but also aboveground in the ecosystems of rice fields. Previous studies speculated that the diversity and functionality of shoot-associated microbes may influence rice health (37, 38). FACE studies also examined geochemical processes such as CH_4_ emission, and found that CH_4_ emission from the rhizosphere to the atmosphere occurred through the shoots of rice plants (9, 43, 54). Schütz *et al.* (53) showed that most of the CH_4_ that was emitted from paddies occurred through rice plants, and Wassmann *et al.* (65) demonstrated that up to 90% of CH_4_ derived from a rice paddy was emitted from rice plants. Nouchi *et al.* (43) found that the concentration of CH_4_ in the medullary cavities of the culm was approximately 2,900-fold higher than that of ambient air under field conditions. Methanotrophs have also been isolated from leaves in several plant species (28, 61), and have been suggested to play a significant role in the aboveground tissues of rice plants (31). Based on a metagenomic analysis of the rice phyllosphere, Knief *et al.* (33) proposed that more methanotrophic bacteria inhabited the aboveground tissues of rice plants than other plants. However, the diversity and ecological functions of the microbial community in the aboveground tissues of rice plants currently remains unclear (22), and a comprehensive study has not yet been conducted on the effects of global warming on the microbial communities in the aboveground tissues of plants.

In the present study, we analyzed the effects of free-air CO_2_ enrichment (FACE), elevated surface water-soil temperature (ET), and low nitrogen (LN) on communities of leaf blade-associated bacteria and leaf sheath-associated bacteria. Our aims were to reveal the characteristics of microbial community structures in the aboveground tissues of rice plants and to clarify the responses of these microbial communities to different environmental factors, which are expected to change during the course of global warming.

## Materials and Methods

### Design of the experimental site, CO_2_ enrichment, surface water-soil warming, and field management

The present study was conducted during the 2011 growing season as a part of the rice-FACE research at Tsukubamirai, Ibaraki, Japan (35°58′27″N, 139°59′32″E, and 10 m a.s.l.). The experimental site was established in 2010, and the climate and experimental design of FACE was described previously by Nakamura *et al.* (42). Briefly, four rice paddy fields were used as replicates, and two treatment areas at ambient levels of CO_2_ (AMBI) and enriched CO_2_ (FACE) were set into each field. Each treatment area was a 240-m^2^ octagon (“ring,” hereafter). The FACE rings used emission tubes on all eight sides at the height of the canopy that released pure CO_2_ from the windward sides to maintain a stable concentration at the center of the ring. The CO_2_ level was set to 200 μmol mol^−1^ above the ambient concentration (42). The AMBI and FACE rings were separated by at least 70 m (center to center), which is considered to be sufficient to prevent cross-contamination by CO_2_ from a FACE ring (19).

Each ring also included surface water-soil temperature treatments in a split-plot design. Under standard nitrogen fertilization, temperatures were normal (ambient temperature, NT) or elevated (2°C above NT, ET) (*i.e.*, AMBI-NT, AMBI-ET, FACE-NT, and FACE-ET plots). Surface water-soil warming was achieved using heating wires placed on the soil surface between the rows with continuously measurements of the water temperature by a Pt100 thermometer (Chino, Tokyo, Japan). In a preliminary experiment, we confirmed that the water and plow-layer temperatures were almost uniformly elevated. The ET plot was enclosed using corrugated PVC panels to restrict the exchange of the paddy water with the surrounding area. The rings also contained low nitrogen plots (LN) with normal surface water-soil temperature (AMBI-LN and FACE-LN plots). The LN plots did not receive nitrogen fertilization. An outline of the experimental design in the present study is summarized in Table 1.

Rice (*Oryza sativa* L. cv. Koshihikari) was sown on 25 April 2011 in seedling trays. On 25 May 2011, seedlings at the five-leaf stage were manually transplanted into the rings with three seedlings per hill. Hills and rows were 15 and 30 cm apart, respectively, with a resultant density of 22.2 hills m^−2^. Fertilizers were applied as a basal dressing. Except in the LN plots, nitrogen was supplied at 8 g N m^−2^ (2 and 6 g N m^−2^ as urea and coated urea, respectively; 4 g of LP-100 and 2 g of LP-140; JCAM-Agri, Tokyo, Japan). Phosphate and potassium were applied as a compound fertilizer (Sumitomo Chemical, Tokyo, Japan) containing 4.36 g P m^−2^ and 8.30 g K m^−2^. The soil was Fluvisol with a mean organic carbon content of 21.4 g kg^−1^ DW and total nitrogen of 1.97 g kg^−1^ DW. Rice straw from the previous year was removed; however, stubble was incorporated into the soil within 1 month of the harvest. All agronomic practices were similar to those of local farmers.

### Growth evaluation and sampling

To clarify how the environmental factors examined affected bacterial community structures, we harvested the aboveground tissues of three plants from each plot in a ring as a composite sample on 5 July 2011 at the panicle initiation stage and immediately transported them on ice to the laboratory. The plants were washed well with tap water and rinsed with sterilized water. The shoots were then separated into leaf blades and sheaths. The samples were stored at −80°C until use. Four composite samples collected from plots of the same treatment in four rings of AMBI or FACE were individually used for DNA preparation and PCR amplification. We also sampled rice plants (6 hills, except for LN plots in which 4 hills were sampled) to determine growth traits, including tiller density and biomass. Soil samples (reduced layer) were simultaneously collected from the plant sampling sites for chemical analyses. The chemical characteristics of the soil were determined by the Tokachi Nokyoren Agricultural Research Institute (Obihiro, Hokkaido, Japan) (Table 2). On the same day of sampling plant materials from fields, the emission of CH_4_ from the paddy soil under the standard nitrogen condition was also measured by a chamber method as described previously (44).

### Clone library construction and sequence analysis

A composite sample of leaf blade tissue (15 g) or leaf sheath tissue (30 g) was homogenized in a blender (Model 911 Clamshell; Hamilton Beach/Proctor-Silex, Southern Pines, NC, USA) without surface sterilization to prepare leaf blade- or leaf sheath-associated bacterial cells (including epiphytes and endophytes), and the bacterial cells were extracted from the tissues and purified by a bacterial cell enrichment method (21). Total DNA from each bacterial cell fraction was extracted and subjected to PCR amplification of the 16S rRNA gene in order to construct a clone library as described previously (21). The PCR products of 4 composite samples collected from plots of the same treatment in rings were combined as a representative sample and were used to construct a clone library. Sequences were examined using OrientationChecker (3). The presence of chimeras was assessed using MALLARD (3). The remaining sequences were aligned using CLUSTAL W (58). Based on these alignments, we constructed a distance matrix using the DNADIST program from PHYLIP ver. 3.66 (http://evolution.genetics.washington.edu/phylip.html) with the default parameters. The resulting matrices were processed using Mothur (51) to generate diversity indexes. Library coverage was calculated with the nonparametric estimator *C* (18). We used the reciprocal of Simpson’s diversity index (1/*D*) to assess the level of dominance in a community (67). UniFrac (34) was applied to examine similarities between clone libraries with the abundance-weighted option.

### Phylogenetic analysis

The phylogenetic composition of the library sequences was evaluated using the LibCompare program of RDP-II release 10 (63), with confidence levels of 80%. BLASTN (2) was also used to classify the clones and identify the closest relatives in public databases. In the phylogenetic analysis, sequences were aligned using CLUSTAL W (58). The neighbor-joining method was used to build a phylogenetic tree (49). The PHYLIP-format tree output was obtained using the bootstrapping procedure (16) with 1,000 bootstrap trials. A tree was constructed using TreeView (45).

### Accession numbers of nucleotide sequences

The nucleotide sequences of 16S rRNA genes for the clone libraries have been deposited in the DDBJ database under the accession numbers shown in Table S1.

## Results

### CO_2_ and temperature control

The average concentration of CO_2_ between 1 June and 5 July was 385 μmol mol^−1^ in the AMBI rings versus 577 μmol mol^−1^ in the FACE rings. The target achievement ratio (TAR), defined as the fraction of time that [CO_2_ ] deviated by <20% (TAR-20) or <10% (TAR-10) for the same period was 95% for TAR-20 and 74% for TAR-10, and were similar to the values observed in 2010 (42). During the same time period, the averages for water temperature just below the surface and soil temperature at a depth of 10 cm were 25.0 and 23.9°C in NT plots and 26.7 and 25.3°C in ET plots, respectively. Surface water-soil temperatures in the LN plots were slightly higher than those in the NT plots (by 0.18°C), and this difference was attributed to more light reaching the soil surface because of the smaller leaf area under the LN condition. The average air temperature between 1 June and 5 July was 22.4°C.

### Rice growth and soil characteristics

No significant differences were observed in soil characteristics between the experimental plots (Table 2). The FACE treatment significantly increased tiller density and total dry mass (*P*<0.05, Table 3). FACE increased total dry mass by 16%, leaf blade mass by 14%, stems and sheaths by 20%, and roots by 10%. Surface water-soil temperature and nitrogen levels also had significant (*P*<0.001) effects on rice growth, except for root mass. As expected, the LN treatment reduced all aboveground biomass more than the the control (AMBI-NT), whereas the ET treatment increased the aboveground biomass. The effects of split-plot factors and CO_2_ were generally additive, as evidenced by no significant interaction. These split-plot effects were most apparent in aboveground tissues in spite of the temperature and nitrogen treatments that were imposed on the soil. The ratios of CH_4_ emission for FACE/AMBI and ET/NT under the standard nitrogen condition were 1.28 and 1.17, respectively, and these values were not significantly different.

### Statistical characteristics of bacterial communities in rice shoots

As expected from the harsh environmental condition of leaf blades, all indexes for bacterial diversity were lower in leaf blades than in leaf sheaths (Table 4). Regarding the environmental factors examined, the reductions observed in diversity indexes in FACE-LN of leaf blade-associated bacteria were more prominent than in the other plots (Table 4). The Shannon and Simpson indexes for leaf blade-associated bacteria were slightly lower in AMBI-ET and FACE-NT than in the control (AMBI-NT), whereas these indexes for leaf blade-associated bacteria in FACE-ET were similar to those in the control. In contrast to the results for leaf blade-associated bacteria, the various environmental conditions had less impact on the diversity indexes of leaf sheath-associated bacteria.

### Overview of bacterial community structures in rice shoots

**Fig. 1:**
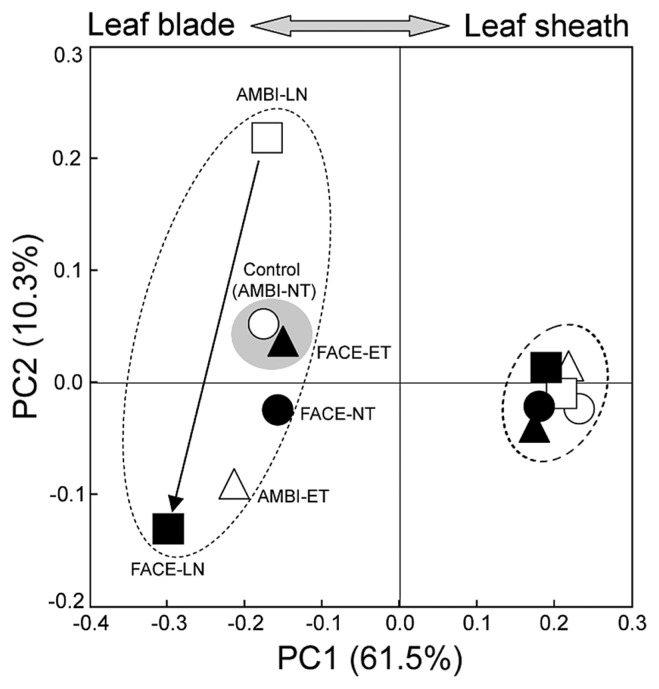
Principal-coordinates analysis of 16S rRNA gene clone libraries of leaf blade- and leaf sheath-associated bacteria under ambient (AMBI) and FACE conditions. The ordination was constructed from all sequence data using UniFrac distances weighted by the relative abundance. NT, normal surface water-soil temperature with standard nitrogen fertilization; ET, elevated surface water-soil temperature (+2°C from NT) with standard nitrogen fertilization; LN, NT with no nitrogen fertilization. The left cluster consists of leaf blade-associated bacteria and the right cluster shows leaf sheath-associated bacteria. The arrow indicates a community shift described in the text. The shaded circle emphasizes the high similarity of community structures between the control (AMBI-NT) and FACE-ET.

Using all sequence data for leaf blade- and leaf sheath-associated bacteria, principal coordinate analyses (PCoAs) revealed that each of these bacterial communities had a distinct structure, as shown by the PC1 (Fig. 1), which was mainly explained by the difference in the relative abundance of *Planctomycetes* (Table 5). These analyses also suggested that the community structures of leaf blade-associated bacteria were more sensitive to environmental changes than those of leaf sheath-associated bacteria. Thus, leaf blade samples were scattered along both PC1 and PC2 directions relative to the control, while all leaf sheath samples (including the control) formed a tighter cluster than that of leaf blade samples (Fig. 1). Among the environmental factors examined, the treatment of no nitrogen fertilizer alone strongly affected the community structures of leaf blade-associated bacteria, as indicated by the large shift from the control (AMBI-NT) to AMBI-LN. The strongest effect of FACE was observed on leaf blade-associated bacteria under low nitrogen conditions, as demonstrated by the community shift from AMBI-LN to FACE-LN (arrow in Fig. 1).

**Fig. 2:**
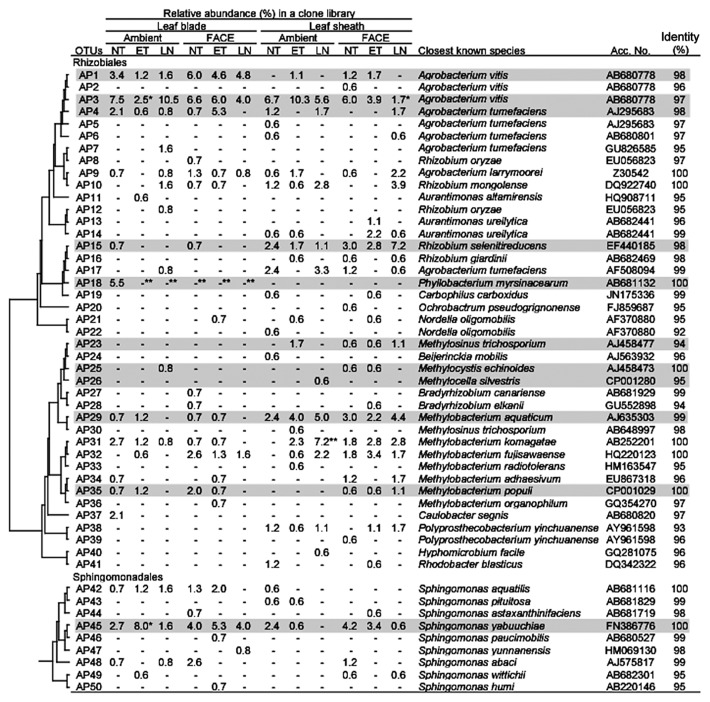
Phylogenetic distribution of OTUs for *Alphaproteobacteria* responding to environmental factors in the 16S rRNA gene clone libraries of rice leaf blade- and leaf sheath-associated bacteria under ambient and FACE conditions. The dendrogram indicates the phylogenetic relationships among the representative sequences of OTUs (defined by ≥97% identity). The table indicates the relative abundance of clones belonging to each OTU in each library and the results of a BLAST search using the representative sequences. ** and * indicate significant differences between the control (ambient CO_2_ with normal temperature and standard nitrogen level) and other samples at *P*<0.01 and *P*<0.05, respectively. Shading indicates OTUs described in the main text.

**Fig. 3:**
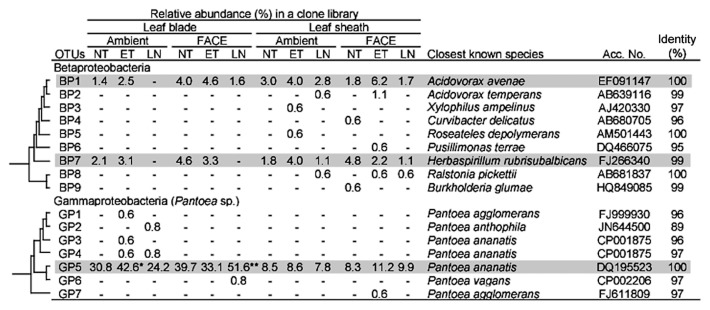
Phylogenetic distribution of OTUs for *Beta-* and *Gammaproteobacteria* responding to environmental factors in the 16S rRNA gene clone libraries of rice leaf blade- and leaf sheath-associated bacteria under ambient and FACE conditions. The dendrogram indicates the phylogenetic relationships among the representative sequences of OTUs (defined by ≥97% identity). The table indicates the relative abundance of clones belonging to each OTU in each library and the results of a BLAST search using the representative sequences. ** and * indicate significant differences between the control (ambient CO_2_ with normal temperature and standard nitrogen level) and other samples at *P*<0.01 and *P*<0.05, respectively. Shading indicates OTUs described in the main text.

An assessment of the phylogenetic composition revealed the unique characteristics of community structures for leaf blade- and leaf sheath-associated bacteria (Tables 5 and S2). At the phylum level, the most dominant population for both leaf blades and leaf sheaths under all conditions was *Proteobacteria* (51.2–88.7% in Table 5). *Proteobacteria* were more abundant in leaf blades than in leaf sheaths, except for the AMBI-LN plot. Within the *Proteobacteria*, *Alpha-* and *Gammaproteobacteria* dominated in both leaf blades and leaf sheaths (15.9–33.7% and 11.3–58.7% in Table 5, respectively). Among *Alphaproteobacteria*, *Rhizobium/Agrobacterium*, *Methylobacterium*, and *Sphingomonas* were the dominant taxa under all conditions, except in the case of leaf sheaths in AMBI-LN (Table S2). The clustering analyses identified two operational taxonomic units (OTUs) in this group: OTU AP3 for *Rhizobium* and OTU AP45 for *Sphingomonas* (Fig. 2). The representative sequences of AP3 and AP45 showed 97% and 100% identities to *Agrobacterium vitis* and *Sphingomonas yabuuchiae*, respectively. Two OTUs of *Alphaproteobacteria* (AP15, AP29), showing high identities to *Rhizobium selenitireducens* and *Methylobacterium aquaticum*, respectively, were more stably present in leaf sheaths than in leaf blades under all conditions examined (Fig. 2). In *Gammaproteobacteria*, OTU GP5, showing an identical sequence to *Pantoea ananatis*, dominated in both leaf blades and leaf sheaths (Fig. 3). The abundance of this OTU was markedly higher in leaf blades (24.2–51.6%) than in leaf sheaths under all conditions examined, indicating the preferential habitation of this OTU for leaf blades.

**Fig. 4:**
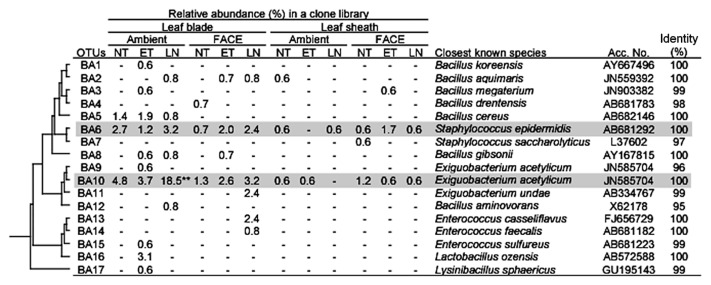
Phylogenetic distribution of OTUs for *Bacilli* responding to environmental factors in 16S rRNA gene clone libraries of rice leaf blade- and leaf sheath-associated bacteria under ambient and FACE conditions. The dendrogram indicates the phylogenetic relationships among the representative sequences of OTUs (defined by ≥97% identity). The table indicates the relative abundance of clones belonging to each OTU in each library and the results of a BLAST search using the representative sequences. ** and * indicate significant differences between the control (ambient CO_2_ with normal temperature and standard nitrogen level) and other samples at *P*<0.01 and *P*<0.05, respectively. Shading indicates OTUs described in the main text.

**Fig. 5:**
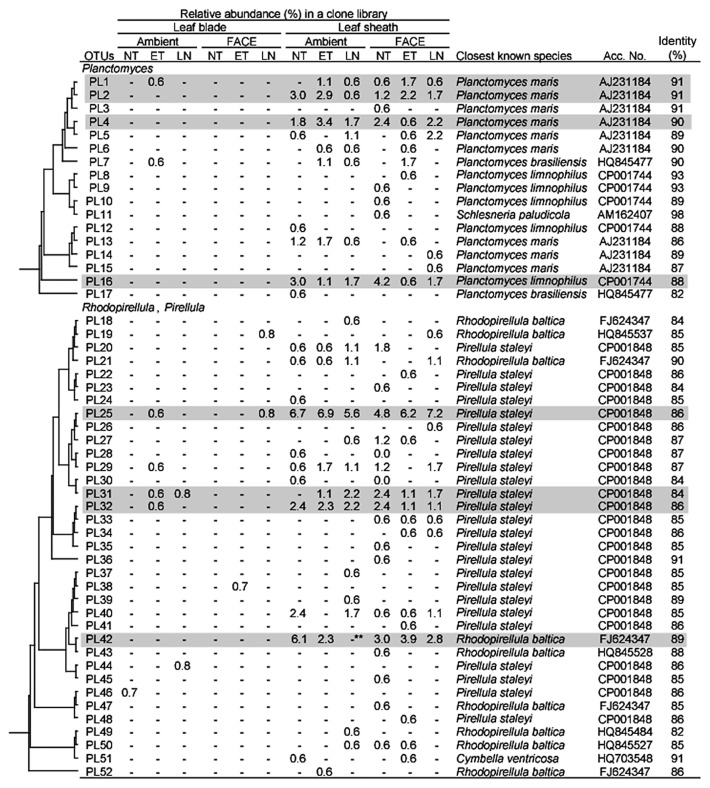
Phylogenetic distribution of OTUs for *Planctomycetes* responding to environmental factors in 16S rRNA gene clone libraries of rice leaf blade- and leaf sheath-associated bacteria under ambient and FACE conditions. The dendrogram indicates the phylogenetic relationships among the representative sequences of OTUs (defined by ≥97% identity). The table indicates the relative abundance of clones belonging to each OTU in each library and the results of a BLAST search using the representative sequences. ** and * indicate significant differences between the control (ambient CO_2_ with normal temperature and standard nitrogen level) and other samples at *P*<0.01 and *P*<0.05, respectively. Shading indicates OTUs described in the main text.

*Firmicutes* was also a dominant taxon in rice shoots (Table 5). Both *Bacilli* and *Clostridia* were more abundant in leaf blades than in leaf sheaths. Within *Bacilli*, *Bacillus*, *Paenibacillus*, *Staphylococcus*, and *Exiguobacterium* were more abundant in leaf blades (Table S2). The clustering analyses identified two dominant OTUs (BA6 and BA10 corresponding to *Staphylococcus* and *Exiguobacterium*, respectively, in Fig. 4). *Clostridia*, which are obligate anaerobic bacteria, were also slightly more abundant in leaf blades (Table 5), which are generally considered to be an aerobic environment due to photosynthesis. *Planctomycetes*, which have rarely been reported in association with the aerial tissues of plants, were exclusively present in leaf sheaths at a high abundance under all conditions examined (20.0–30.5% in Table 5). Although the *Planctomycete* community in rice leaf sheaths appeared to be phylogenetically diverse, relatively dominant OTUs were identified by clustering analyses (Fig. 5). In the genus *Planctomyces*, OTUs PL1, PL2, PL4, and PL16 were detected under most environmental conditions examined, except for OTU PL1 in AMBI-NT. In the genus *Pirellula*, OTUs PL25, PL31, and PL32 were detected under most conditions examined, except for OTU 31 in AMBI-NT. OTU PL25 was identified as the most dominant and stable OTU in the *Planctomycete* community (Fig. 5). A prominent feature of this community in rice leaf sheaths was that the representative sequences of 16S rRNA genes for most OTUs had less than 95% identity to known species (Fig. 5). A phylogenetic tree analysis revealed the presence of three unique clusters for rice leaf sheaths (clusters A–C in Fig. 7). Clusters A and B were distantly related to the known species of two genera, *Pirellula* and *Rhodopirellula*, whereas cluster C was located within the cluster of known *Planctomyces* species.

### Impacts of environmental factors on leaf blade-associated bacteria

**Fig. 6:**
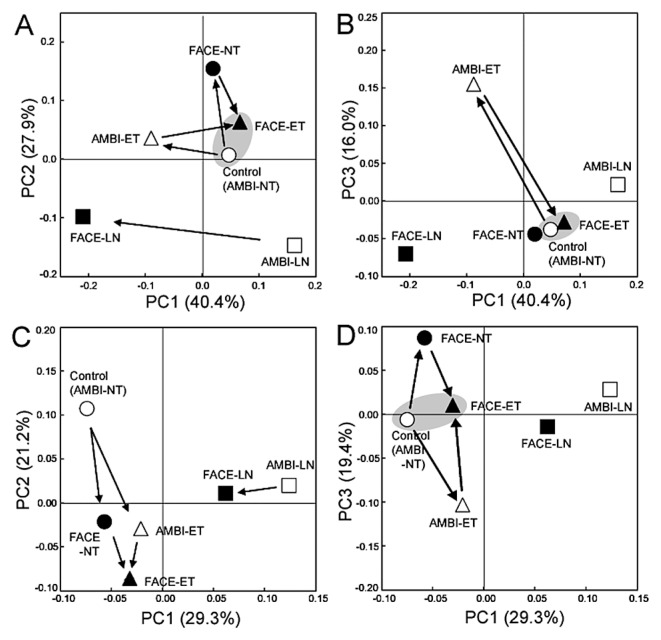
Principal-coordinates analysis of 16S rRNA gene clone libraries of rice shoot-associated bacteria under ambient (AMBI) and FACE conditions. The ordinations were separately constructed for leaf blades (A, B) and leaf sheaths (C, D) using UniFrac distances weighted by the relative abundance. NT, normal surface water and soil temperature with standard nitrogen fertilization; ET, elevated surface water and soil temperature (+2°C from NT) with standard nitrogen fertilization; LN, NT with no nitrogen fertilization. The arrow indicates a community shift described in the text. The shaded circle emphasizes the high similarity of community structures between the control (AMBI-NT) and FACE-ET.

**Fig. 7:**
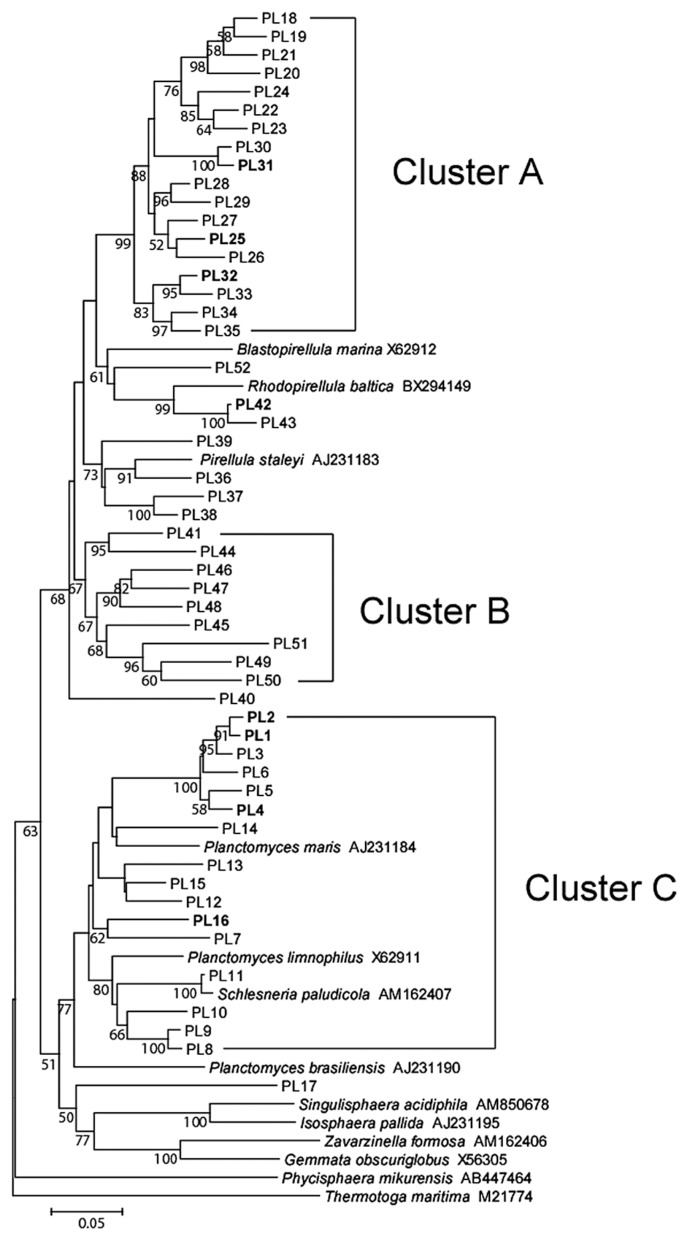
Phylogenetic tree of rice shoot-associated *Planctomycetes* based on representative sequences of OTUs in clone libraries of 16S rRNA genes. The tree was constructed by the neighbor-joining method. The scale represents 0.05 substitutions per site. The numbers at the nodes are the proportions of 1,000 bootstrap resamplings, and values <50% are not shown. The OTUs described in the main text are indicated in bold.

UniFrac PCoA showed that the bacterial community in leaf blades was affected more by ET than by FACE as a single environmental factor, as shown by the degree of the community shift from the control (AMBI-NT) to AMBI-ET along PC1 and PC3 (40.4% and 16.0%, respectively, in Fig. 6A and 6B) relative to FACE-NT (Fig. 6A). In contrast, almost no shift was observed from the control to FACE-NT and FACE-ET along PC1 and PC 3 (Fig. 6B). These results indicated that the individual effects of the ET and FACE treatments on leaf blade-associated bacteria were qualitatively and quantitatively different from those of the combined FACE and ET treatments, whereas the community structure of leaf blade-associated bacteria in the combined FACE and ET treatments was shown to be highly similar to that in the control (Fig. 6A and 6B).

The community structure of leaf blade-associated bacteria under low nitrogen conditions was distinct from those under standard nitrogen fertilization, as shown by the shifts along PC2 (Fig. 6A). The greatest impact of FACE was observed on leaf blade-associated bacteria under low nitrogen conditions, as shown by the community shift from AMBI-LN to FACE-LN along PC1 (Fig. 6A and 6B). PC1 in Fig. 6A can be explained by population shifts in *Rhizobium* and *Pantoea* (Fig. 6).

Phylogenetic analyses revealed that the abundance of *Proteobacteria* and *Firmicutes* in FACE-NT (88.7% and 7.9% respectively) differed significantly from that in the control (74.0% and 17.1%, respectively, in Table 5). In FACE-NT, the abundance of three classes of *Proteobacteria* (*Alpha-*, *Beta-*, and *Gammaproteobacteria*) was higher than that in the control and, conversely, the abundance of two classes of *Firmicutes* (*Bacilli* and *Clostridia*) was lower. As expected from the results of UniFrac PCoA, the phylogenetic composition of leaf blades in FACE-ET was highly similar to that in the control at the phylum and class levels (Table 5).

The relative abundance of *Alphaproteobacteria* was significantly lower in AMBI-ET and FACE-LN (19.1% and 15.9% respectively) than in the control (30.8%) (Table 5). Clustering analyses revealed that the abundance of three OTUs (AP1, AP3, and AP4) for *Agrobacterium* species decreased in AMBI-ET (Fig. 2). The abundance of *Methylobacterium* was lower in leaf blades treated with LN (0.8% for AMBI-LN, 1.6% for FACE-LN) than in the control (4.8%) (Table S2), and the number of OTUs for *Methylobacterium* was lower in LN under both AMBI and FACE conditions (1 OTU for each) than in the control (4 OTUs in Fig. 2). *Phyllobacterium* represented by OTU AP18 was only detected in the leaf blades of the control (Fig. 2).

Similar to the population shifts in *Methylobacterium*, the abundance of two OTUs for *Betaproteobacteria* in leaf blades was also lower in LN under both AMBI and FACE conditions (0% and 1.6%, respectively) than in the control (3.4%) (Table 5). The abundance of *Gammaproteobacteria* exclusively represented by OTU GP5 (*Pantoea* sp.) was higher in AMBI-ET and FACE-LN (42.6% and 51.6%, respectively) than in the control (30.8%) (Fig. 3). *Firmicutes* was shown to be sensitive to nitrogen fertilization levels as indicated by the increase observed in the relative abundance of *Bacilli*, especially OTU BA10 belonging to the genus *Exiguobacterium*, in AMBI-LN and FACE-LN (Table 5 and Fig. 4).

### Impacts of environmental factors on leaf sheath-associated bacteria

As was the case in leaf blade-associated bacteria, PCoA clearly indicated that the most dominant effect on the bacterial community in leaf sheaths was nitrogen fertilization, as shown by PC1 (29.3% in Fig. 6C), and this results was attributed to population shifts in *Methylobacterium* and *Planctomycetes* (Table S2). Under low nitrogen conditions, FACE was shown to have less impact on the community of leaf sheath-associated bacteria than on that of leaf blade-associated bacteria, as indicated by the community shifts from AMBI-LN to FACE-LN in Fig. 1. The individual effects of ET and FACE caused similar directional community shifts from AMBI-NT (the control) to AMBI-ET and FACE-NT in leaf sheaths along PC2 (21.2%), and the community structure of leaf sheath-associated bacteria in FACE-ET differentiated even further from the control (Fig. 6C). However, the community structure in FACE-ET was more similar to the control along PC3 than to those in AMBI-ET and FACE-NT, as observed for leaf blade-associated bacteria (Fig. 6A), indicating that a part of the community structure of leaf sheath-associated bacteria in FACE-ET shared some similarity to that in AMBI-NT.

The abundance of *Methylobacterium* was higher in all environmental treatments, (6.9–14.4%) than in the control (2.4%) (Table S2), and the number of OTUs for *Methylobacterium* was also higher (three to five OTUs) than that in the control (one OTU) (Fig. 2). The abundance of *Sphingomonas* was affected more by no nitrogen fertilization under both the AMBI and FACE conditions (0% and 1.1%, respectively) than the control (3.7%) (Table S2). The abundance of *Planctomycete* was markedly lower in AMBI-LN (20.0%) than in the control (30.5%), and was partially explained by a decrease in *Rhodopirellula*, corresponding to OTU PL42 (Fig. 5). In contrast, the populations of two dominant genera (*Planctomyces* and *Pirellula*) were relatively stable under all conditions examined (Table S2).

## Discussion

The present study clarified some characteristics of bacterial communities in the aerial tissues of rice plants, as well as the impacts of their community structures in response to environmental factors under field conditions. The distinct tissue specificity of rice plant-associated bacteria was shown for leaf blades and sheaths and the community structures of leaf blade-associated bacteria were shown to be more sensitive to environmental stresses than those of leaf sheath-associated bacteria (Fig. 1). Similar results were observed in the metabolic statuses of leaf blades and leaf sheaths, as assessed by NMR-based metabolic profiling (Fig. S1). Thus, the distinct separation of NMR profiles for leaf blade and leaf sheath samples along with PC2 in Fig. S1-A could explain the tissue specificity of shoot-associated bacteria because such bacteria may depend highly on plant metabolites as a nutrient source. The effects of FACE were more clearly observed in leaf blade samples than in leaf sheath samples, similar to the bacterial community analyses. Moreover, detailed analyses for metabolite components by loading plots revealed that one of the main effects of FACE on the metabolic statuses of both leaf blades and leaf sheaths appeared to be a shift in the sucrose content, which could be an important potential carbon source for microbes (Fig. S1-B). These results suggested that the diversity of symbiotic bacteria eventually reflected the physiological status of rice plants and that the tissue specificity of metabolites, such as different sugar contents, was a dominant force over the environmental factors examined for shaping the bacterial community structures in rice shoots. UniFrac PCoA revealed that the bacterial community in leaf blades was affected more by ET than by FACE as a single environmental factor (Fig. 6), as was the case in root-associated bacteria (44). In the present study, the combined treatments of FACE and ET under standard nitrogen fertilization led to similar community structures to the control (Fig. 6A and 6D), whereas ET and FACE had distinct impacts on bacterial communities in the aerial tissues of the rice plant (Figs. 1 and 6). The impacts of the combined treatments of FACE and ET on plant biomass were found to be additive (Table 3). These results suggest that realistic experimental settings need to be considered in order to assess the impacts of environmental factors in an open field. The most prominent impact of FACE was observed in the communities of leaf blade-associated bacteria under low nitrogen conditions (shift from AMBI-LN to FACE-LN in Figs. 1 and 6), which may be explained by elevated CO_2_ levels lowering leaf nitrogen concentrations and accelerating leaf senescence (1, 32). Hence, elevated CO_2_ levels may have a significant microbiological impact on rice plants in which the input of nitrogen fertilization is low or on a plant communities in natural wetland.

The phylogenetic analyses revealed that *Proteobacteria*, especially *Alpha-* and *Gammaproteobacteria*, was the dominant phylum in both leaf blade- and leaf sheath-associated bacteria, (Table 5). The dominance of *Alpha-* and *Gammaproteobacteria* in aboveground tissues has been reported for some plant species that are phylogenetically distantly related to *O. sativa* (20, 23, 24, 56, 60), suggesting that these two phyla could be core community members in the aboveground tissues of plants. Three genera in *Alphaproteobacteria* (*Rhizobium/Agrobacterium*, *Methylobacterium*, and *Sphingomonas*), which have been identified as dominant taxonomic groups in stem and leaf tissues (20, 22, 33, 60), were also dominant in the leaf blade and sheath tissues of the rice plant (Table S2). Within *Alphaproteobacteria*, two OTUs showing high similarities to *A. vitis* and *S. yabuuchiae*, (AP3 and AP45, respectively) were highly abundant and persistent in leaf blades and sheaths (Fig. 2). Among the *Gammaproteobacteria*, OTU GP5, the representative sequence of which was identical to *P. ananatis*, was dominant in aboveground rice tissues (Fig. 3). These *Alpha*- and *Gammaproteobacteria* have been isolated from the seeds, leaves, and stems of rice plant (7, 38, 66). The distribution of these taxa across the phylogenetically wide range of plant species with high persistence and abundance (20, 23, 24, 56, 60) suggests the ecological importance of these bacteria in a phytosphere.

In the leaf blade, FACE under the 3 environmental treatments (FACE-NT, FACE-ET, and FACE-LN) generally increased the abundance of *Proteobacteria* above that in the control (AMBI-NT) (Table 5). Similar results were observed for the increase in biomass production by FACE (Table 3). This FACE effect was also partially supported by an increase in certain metabolites such as the sucrose (Suc) content in leaf blades in FACE (Fig. S1-B).

Within *Alphaproteobacteria*, *Rhizobium*/*Agrobacterium* and *Methylobacterium* in leaf blades were highly sensitive to environmental changes (Fig. 2). These genera are known to be beneficial bacteria in several plant species including *O. sativa* (7, 35). In addition, the *Gammaproteobacteria* population was exclusively represented by an OTU that is identical to *P. ananatis* (GP5 in Fig. 3), which has been recognized worldwide as an emerging plant pathogen for many crops, including rice (13). This species causes rice grain and sheath rot (10) as well as rice leaf blight (41). Several studies reported the potential threat of this pathogen on stable rice production in hot and humid environments (8, 11, 12). Therefore, the bacterial groups described above need to be carefully monitored in future studies when assessing the effects of environmental changes, including global warming.

*Firmicutes*, including *Bacilli* and *Clostridia*, was also a more dominant phylum in leaf blades than in leaf sheaths (Table 5). This result may have been due to Gram-positive bacteria being physically more resistant to environmental factors such as light and dry stresses than Gram-negative bacteria and also adaptive to leaf blades, which is a more severe environment than leaf sheaths. Alternatively, bacterial groups in *Firmicutes* may form an anaerobic nitrogen-fixing consortia (ANFICOs) consisting of nitrogen-fixing *Clostridia* and diverse nondiazotrophic aerobic bacteria, which are often bacillus or other aerobic bacteria in *Bacilli*, in order to overcome a low nitrogen status by the fixation of atmospheric nitrogen (40). In *Bacilli*, populations of two OTUs showing high similarities to *S. epidermis* and *E. acetylicum* (OTUs BA6 and BA10, respectively in Fig. 4) were stably present in leaf blades under all conditions, except that *Exiguobacterium* sp. was significantly increased in AMBI-LN (Table S2). The relationship between *Exiguobacterium* sp. and plants has been reported previously (57, 64). *Staphylococcus epidermis* is also a common member of the phytosphere community of both above- and belowground plant tissues (6, 21), and has been attracting attention as an opportunistic human pathogen (5). The community analyses revealed that *Clostridia*, which are obligate anaerobic bacteria, were more abundant in leaf blades, which are considered to be a more aerobic environment due to photosynthesis, than leaf sheaths (Table 5 and Table S2). Minamisawa *et al.* (40) showed the presence of *Clostridia* in the aerial tissues of many gramineous plants, and proposed the concept of ANFICOs. Saito *et al.* (48) subsequently reported the presence of ANFICOs in diverse plant species, including *O. sativa*. As shown in the present study, such syntrophic relationships between anaerobic and aerobic microbes in aerial plant tissues may be more common than previously thought.

Another unexpected result of the present study was the abundant and stable presence of *Planctomycetes* in leaf sheaths (Table 5). Derakshani *et al.* (14) described the relationship between *Planctomycetes* (specifically, *Pirellula*) and rice roots. However, few studies have successfully identified a relationship between *Planctomycetes* and aerial plant tissues (*e.g.*, Wang *et al.*, 2008[62]). Sessitsch *et al.* (55) isolated a 16S rRNA gene clone showing 94% identity to *Pirellula* from potato stems. We generally found a negative relationship between the abundance of *Methylobacterium* and *Rhodopirellula* (Table S2). These genera may have a competitive ecological relationship because the genomic analysis of *Rhodopirellula baltica* revealed the presence of genes encoding proteins highly similar to the tetrahydromethanopterin-dependent enzymes involved in C1 metabolism (17). While, proteins related to methanol-based methylotrophy linked to *Methylobacterium* were highly abundant in the phyllosphere (33). To date, methylotrophy has not been experimentally proven in members of the *Planctomycetes*. However, a codon usage analysis suggested a high level of gene expression related to C1 metabolism in *Planctomycetes* (30).

Knief *et al.* (33) demonstrated that methanotrophic *Alpha-* and *Gammaproteobacteria* were detectable in the phyllosphere using PCR targeting a subunit of the membrane-bound methane monooxygenase (*pmoA*) and also cultures; however, they found no evidence for the presence of methane monooxygenase in the phyllospheric metaproteome or encoding genes in the metagenome of the rice phyllospheric microbiota. Furthermore, the presence of methanotroph groups in aerial tissues has already been reported in other plant species (15, 28). The present study also identified the typical Alphaproteobacterial methanotrophs in the aerial tissues of rice, especially in the leaf sheath (OTUs AP23, AP25, and AP26 in Fig. 2). In metabolic profiling, the leaf sheath was characterized by a lower total metabolite concentration and relatively higher proportion of Suc, fructose (Fru), glucose (Glc), and formate (Fig. S1-B). The higher proportion of formate, which could be a product of C1 metabolism, may affect the observed distribution of the methanotrophs in leaf sheaths. Since bacterial cells were physically extracted from plant tissues, the bacterial cells of slow growers, such as Alphaproteobacterial methanotrophs, may not be efficiently extractable for technical reasons and their abundance in plant tissues could be underestimated in evaluations performed with current methodologies.

In conclusion, our findings provide basic characteristics of bacterial diversities in rice leaf blades and sheaths under field conditions. Using a FACE facility in Japan, we also assessed the impacts of environmental factors on rice shoot-associated bacterial communities. Our analyses revealed that the combined effects of environmental factors (*e.g.*, CO_2_ level and temperature) and management practices (*e.g.*, nitrogen fertilization) need to be examined as a realistic model ecosystem to assess the effects of global warming rather than conducting an assessment on an impact of a single environmental factor. The results of the community analyses in the present study also indicated that the leaf blade-associated microbial community may be used a sensitive indicator for assessing the impacts of environmental factors on both the above- and belowground tissues of rice plants.

## Supplementary Information



## Figures and Tables

**Table 1:** Experimental design of free-air CO_2_ enrichment, elevated surface water-soil temperature and low nitrogen

Atmosphere	Ambient			FACE^a^		
		
Temperature	Ambient	Elevated^b^	Ambient	Ambient	Elevated	Ambient
						
Fertilization	Standard	Standard	Low nitrogen^c^	Standard	Standard	Low nitrogen
						
Plot name	AMBI-NT	AMBI-ET	AMBI-LN	FACE-NT	FACE-ET	FACE-LN

a Rice plants grown under the free-air CO_2_ enrichment condition.

b Rice plants grown in elevated surface water-soil temperature (2°C above abient temperature).

c Rice plants grown with no nitrogen fertilization.

**Table 2:** Chemical characteristics of bulk soil sampled^a^

Plot name^b^	NO_3_ -N^c^	NH_4_ -N^c^	Total N (%)	Total C (%)	pH (H_2_ O)
AMBI-NT	5.0±4.3^c^	36.9±37.3	0.20±0.02	1.8±0.2	5.9±0.2
AMBI-ET	2.7±0.6	34.4±10.6	0.19±0.03	1.9±0.2	5.9±0.2
AMBI-LN	6.3±0.6	11.5±2.1	0.17±0.04	1.6±0.4	5.9±0.1
FACE-NT	6.6±3.8	38.6±12.2	0.22±0.03	2.1±0.3	5.9±0.2
FACE-ET	3.5±1.7	35.7±6.9	0.22±0.04	2.1±0.4	5.9±0.1
FACE-LN	4.8±4.4	21.3±12.3	0.20±0.02	2.0±0.2	5.9±0.1

a The average of 4 replicated plots±standard deviation.

b Sample name stands for the combination of following environmental factors; AMBI, ambient CO_2_ ; FACE, free air CO_2_ enrichment; NT, normal surface water-soil temperature; ET, elevated surface water-soil temperature; LN, no nitrogen fertilization. See the main text for the detailed description for abbreviations of environmental factors. Soil samples were taken from depths of 1–10 cm.

c μg g^−1^ dry soil.

**Table 3:** Tiller density and dry mass by organs, root:shoot ratio and total dry mass of rice plants measured at around the panicle initiation stage (July 5) in the normal surface water-soil temperaure (NT), elevated surface water-soil temperature (ET) and low nitrogen (LN) plots nested in the CO_2_ treatments (ambient and FACE).

Split-plot-factor	Tiller density (/m^2^)			Leaf blade dry mass (g/m^2^)			Stem & sheath dry mass (g/m^2^)			Root dry mass (g/m^2^)			Root:shoot ratio			Total dry mass (g/m^2^)		
											
	Ambient	FACE	Sig^a^	Ambient	FACE	Sig^a^	Ambient	FACE	Sig^a^	Ambient	FACE	Sig^a^	Ambient	FACE	Sig^a^	Ambient	FACE	Sig^a^
AT	471	554	b	112	131	b	138	164	b	54	58	a	0.22	0.20	a	304	353	b
ET	535	586	b	139	157	c	158	192	c	59	65	a	0.20	0.19	a	355	414	c
LN	393	422	a	84	94	a	110	132	a	54	60	a	0.28	0.26	b	248	286	a
Analysis of Variance Results																		
CO_2_	+ (0.082)			+ (0.063)			*			ns (0.186)			ns (0.31)			*		
Split-factor (S)	***			***			***			ns			**			***		
CO_2_ x S	ns			ns			ns			ns			ns			ns		

a The split-plot factors followed by the same letters in each column were not significantly different (*P*>0.05) by the Tukey’s method.

*,**,*** indicate that the effects were significant at *P*<0.05, *P*<0.01 and *P*<0.001, respectively.

**Table 4:** Statistical characteristics of 16S rRNA gene clone libraries derived from rice shoot-associated bacteria under various environmental conditions

Tissues	Leaf blade						Leaf sheath					
		
Growth air conditions	Ambient^a^			FACE^b^			Ambient			FACE		
				
Plots	AT^c^	ET^d^	LN^e^	AT	ET	LN	AT	ET	LN	AT	ET	LN
Statistics												
No. of sequences	146	162	124	151	151	126	164	174	180	168	178	181
No. of OTUs (≥97% identity)^f^	47	50	44	46	49	32	77	76	78	81	82	82
No. of singletons	29	34	30	32	32	19	52	50	50	55	58	54
Library coverage (%)^g^	80.1	79	75.8	78.8	78.8	84.9	68.3	71.3	72.2	67.3	67.4	70.2
Diversity indexes												
Chao1	114.7	130.1	98.4	128.7	119.9	74.8	209.6	187.4	180.0	216.0	288.6	241.0
ACE	148.9	179.5	105.6	290.7	224.8	60.5	332.3	289.3	367.6	397.8	398.7	280.1
Shannon index (*H*′)	3.0	2.7	2.9	2.7	3.0	2.2	3.9	3.8	3.9	4.0	3.9	4.0
Simpson index (1/*D*)	9.1	5.2	9.6	5.9	8.1	3.7	40.5	33.1	39.3	46.1	36.2	37.5

a Rice plants grown under ambient atmosphere condition.

b Rice plants grown under the free-air CO_2_ enrichment condition.

c Rice plants grown in ambientl surface water-soil temperature with standard nitrogen fertilization.

d Rice plants grown in elevated surface water-soil temperature (2°C above AT) with standard nitrogen fertilization.

e Rice plants grown in ambient surface water-soil temperature with no nitrogen fertilization.

f OTUs were defined at ≥97% sequence identity.

g Coverage (*C_x_*) = 1 − (*n_x_*/*N*), where *n_x_* is the number of singletons that are encountered only once in a library and *N* is the total number of clones.

**Table 5:** Phylogenetic compositions of 16S rRNA gene clone libraries derived from leaf blade- and leaf sheath-associated bacteria in rice plants cultivated under different environmental conditions

	Relative abundance in a clone library (%)^a^											
	
Tissues	Leaf blade						Leaf sheath					
		
Atmospheric conditions	AMBI^b^			FACE^c^			AMBI			FACE		
				
Temperature	NT^d^	ET^e^	LN^f^	NT	ET	LN	NT	ET	LN	NT	ET	LN
Proteobacteria	74.0	71.0	57.3**	88.7**	77.5	76.2	51.8	58.0	61.7	51.2	59.0	59.7
Alphaproteobacteria	30.8	19.1*	24.2	33.1	31.1	15.9**	29.9	29.9	32.8	29.8	29.2	33.7
Betaproteobacteria	3.4	5.6	—	8.6	7.9	1.6	4.9	9.2	5.0	7.7	10.7*	3.3
Gammaproteobacteria	37.7	46.3	32.3	45.0	37.1	58.7**	14.0	13.8	22.2	11.3	15.2	18.2
Deltaproteobacteria	2.1	—	0.8	—	—	—	0.6	3.4	1.7	2.4	2.8	1.1
Planctomycetes	1.4	3.7	—	0.7	1.4	0.8	30.5	27.6	20.0*	25.6	22.5	22.6
Firmicutes	17.1	19.2	36.3**	7.9*	13.9	15.1	4.2	1.2	2.8	5.4	6.1	3.6
Bacilli	11.6	15.5	27.4**	6.0	8.6	13.5	1.8	0.6	1.7	3.0	3.3	1.7
Clostridia	5.5	3.7	8.9	2.0	5.3	1.6	2.4	0.6	1.1	2.4	2.8	2.2
Actinobacteria	2.7	0.6	3.2	2.0	4.0	0.8	0.6	1.7	1.1	1.8	—	1.7
Verrucomicrobia	0.7	0.6	0.8	—	—	—	1.8	2.9	2.2	3.0	2.2	1.7
Acidobacteria	—	—	—	—	—	1.6	1.2	0.6	1.7	1.8	—	0.6
Cyanobacteria/Chloroplast	—	—	—	—	0.7	—	0.6	—	—	—	—	—
Chloroflexi	—	—	0.8	0.7	—	—	—	—	—	—	—	—
BRC1	—	—	—	—	—	—	—	—	—	0.6	—	—
Unclassified bacteria	4.1	4.9	1.6	—	2.6	5.6	9.1	8.0	10.6	10.7	10.1	8.3

a* and ** indicate statistical significance at the 1 and 5% levels (*P* < 0.01 and *P* < 0.05), respectively, calculated with the Library Compare of RDP II, between the control (AMBI-NT) and other samples.

b Rice plants grown under ambient atmosphere condition.

c Rice plants grown under the free-air CO_2_ enrichment condition.

d Rice plants grown in normal surface water-soil temperature with standard nitrogen fertilization.

e Rice plants grown in elevated surface water-soil temperature (2°C above NT) with standard nitrogen fertilization.

f Rice plants grown in normal surface water-soil temerature with low nitrogen fertilization.
