# Supplementary material for: Characterization of Leaf Blade- and Leaf Sheath-Associated Bacterial Communities and Assessment of Their Responses to Environmental Changes in CO2, Temperature, and Nitrogen Levels under Field Conditions
Source: Microbes Environ. 2015 Feb 4;30(1):51–62. doi: 10.1264/jsme2.ME14117 (PMC4356464; doi:10.1264/jsme2.ME14117)
Supplement: Supplementary file 1 [file 30_51_s1.pdf]

Supplementary information for NMR based metabolic profiling

Characterization of Leaf Blade– and Leaf Sheath–Associated Bacterial Community and Assessment of Their Responses to Environmental Changes of CO_2, Temperature, and Nitrogen Levels under Field Conditions

SEISHI IKEDA[1] TAKESHI TOKIDA[2], HIROFUMI NAKAMURA[3], HIDEMITSU SAKAI[2], YASUHIRO USUI[2], TAKASHI OKUBO[4], KANAKO TAGO[2], KENTARO HAYASHI[2], YASUYO SEKIYAMA[5], HIROSHI ONO[5], SATORU TOMITA[5], MASAHITO HAYATSU[2], TOSHIHIRO HASEGAWA[2], and KIWAMU MINAMISAWA[4*]

[1] *Memuro Research Station, Hokkaido Agricultural Research Center, National Agriculture and Food Research Organization, Shinsei, Memuro–cho, Kasai–gun, Hokkaido 082–0081, Japan;* [2] *National Institute for Agro–Environmental Sciences, 3–1–3 Kannondai, Tsukuba, Ibaraki 305–8604, Japan;* [3]*Taiyo-Keiki Co., Ltd., 1-12-3 Nakajujo, Kita, Tokyo, 114-0032 Japan;* [4]*Graduate School of Life Sciences, Tohoku University, 2–1–1 Katahira, Aoba–ku, Sendai, Miyagi 980–8577, Japan;* [5]*National Food Research Institute, National Agriculture and Food Research Organization, 2-1-12 Kannondai, Tsukuba, Ibaraki 305-8642, Japan;*

*Corresponding author. E–mail: kiwamu@ ige.tohoku.ac.jp; Tel: +81–22–217–5684; Fax: +81–22–217–5684.

Chemicals

Methanol-d_4 (MeOD, 99.8% D) and 4-(2-hydroxyethyl)-1-piperazineethanesulfonic acid (HEPES)-d_{18} (98% D) were purchased from Cambridge Isotope Laboratories (Andover, MA, USA); deuterium oxide (99% D) was obtained from Sigma-Aldrich (St. Louis, MO, USA).

Sample preparation

The rice plant samples (cv. Koshihikari) for NMR-based metabolic profiling were also harvested at approximately the same growing stage and circadian phase as those of bacterial community structure analysis (July 8, 2011, at 9:00-11:00 AM). Three biological replicates for each experimental design (NT, ET and LN of FACE and AMBI) were collected from a rice paddy field. The aboveground parts of the 18 rice plants were frozen immediately in liquid nitrogen and lyophilized in a FD-20BU/SK01 freeze-dryer (Nihon Techno Service, Chiba, Japan). The first, second and third developed leaves were separated into leaf blade and leaf sheath, respectively. The each organ was ground into powder with a MultiBeads Shocker (Yasui Kikai, Osaka, Japan) and the powder of

each sample (6 mg) was suspended in 300 μL of MeOD/HEPES-d_{18} buffer, which was prepared as described previously[1,2]. The mixture was heated at 50°C for 5 min while shaking at 1400 rpm in a Thermomixer comfort (Eppendorf AG, Hamburg, Germany) and then centrifuged (21,500 ×g, 5 min). The supernatant was used for the NMR experiments.

NMR spectroscopy

Sample solutions were transferred into 3.0 mm O.D. × 103.5 mm NMR tubes (Norell, Landisville, NJ). NMR spectra were recorded on an Avance-500 spectrometer (Bruker BioSpin, Karlsruhe , Germany) equipped with a CryoProbe for 5-mm sample diameters operating at 500.23 MHz for ^{1}H and 125.80 MHz for ^{13}C. The temperature of all NMR samples was maintained at 298 K. The chemical shifts were referenced to the TMS group of sodium 2,2-dimethyl- 2-silapentane-5-sulfonate (DSS, 0.5 mM in MeOD/HEPES-d_{18}) internal standard.

^{1}H NMR spectra were collected using the Bruker pulse program zgpr, and the following acquisition parameters were used: spectral width, 13 ppm; acquisition mode, sequential quadrature detection; offset frequency, 4.9 ppm; the proton 90° pulse, 16.3 μs and 15.0 μs for carbon; relaxation delay, 10 s; number of scans, 128. ^{1}H–^{13}C heteronuclear quantum coherence (HSQC) spectra were collected using echo/antiecho gradient selection (the hsqcetgpsisp pulse program in the Bruker library) with the following parameters: 90° pulse values, 16.3 μs for proton; relaxation delay, 2 s; spectral width, 130 ppm (f1) and 10 ppm (f2); data points, 256 increments of 2048; scans, 64. The chemical shifts were referenced to the methyl group of a DSS internal standard (0.00 ppm for ^{1}H and ^{13}C).

Data analysis

The ^{1}H NMR spectra were processed using the TopSpin software (ver. 3.2, Bruker BioSpin). For principal component analysis (PCA), datasets were generated by subdividing spectra (10.00–0.50 ppm) into integrated regions of 0.04 ppm each using the Amix software (ver. 3.9.14, Bruker BioSpin). The integrated data were then normalised to total intensity of all the variables. The residual CH_2DOD and DSS signals were excluded before further analysis. PCA were performed using the SIMCA software (ver. 13.0.3.0, Umetrics, Umeå, Sweden), and Pareto scaling was applied to PCA.

Annotation of signals

The ^{1}H–^{13}C HSQC spectra were processed with NMRPipe and analyzed using

NMRDraw[3]. Metabolite signals were annotated using a semi-automated annotation program, SpinAssign (http://prime.psc.riken.jp/). Candidate metabolites for each peak were selected from standard compounds by comparing the chemical shift difference. A compound was selected when the chemical shift difference between the standard and queried peak was less than 0.03 ppm and 0.53 ppm for ^{1}H and ^{13}C, respectively.

Supplementary results

PCA was carried out using the datasets containing all 224 variables from the ^{1}H NMR spectra. Score and loading plots of leaf blade and sheath are shown in Figure S1. The score plot reflects the relative proportion of metabolites since each variable was scaled to the total intensity of the corresponding spectrum. The first and second principle components (PC1 and PC2) describes the effect of CO_2 concentration and difference in organ, respectively. The variation on PC1 was explained primarily on the differential abundance of sucrose (Suc) and glycerolipids (GLs) between ambient and FACE condition; leaves grown under FACE condition were characterised by higher levels of Suc, and lower amounts of GLs. Comparison of the raw spectra, that were normalized to the intensity of DSS also confirmed such tendency of increasing and decreasing amounts of Suc and GLs, respectively (data not shown). The variation along PC2 was due to differences in the relative proportion of Suc, fructose (Fru), glucose (Glc), and formate. The raw spectra showed that the total metabolite concentration was lower in leaf sheath than leaf blade, and the metabolite profile in sheath was mainly characterised by relatively higher proportion of the sugars and formate. No clear effects of leaf age and other experimental conditions (NT, ET, and LW) were found on the metabolite levels.

Supplementary references

1 Sekiyama, Y., Chikayama, E. & Kikuchi, J. Evaluation of a Semipolar Solvent System as a Step toward Heteronuclear Multidimensional NMR-Based Metabolomics for C-13-Labelled Bacteria, Plants, and Animals. *Analytical Chemistry* **83**, 719-726, doi:Doi 10.1021/Ac102097u (2011).

2 Sekiyama, Y., Chikayama, E. & Kikuchi, J. Profiling Polar and Semipolar Plant Metabolites throughout Extraction Processes Using a Combined Solution-State and High-Resolution Magic Angle Spinning NMR Approach. *Analytical Chemistry* **82**, 1643-1652, doi:Doi 10.1021/Ac9019076 (2010).

3 Delaglio, F. *et al.* Nmrpipe - a Multidimensional Spectral Processing System Based on Unix Pipes. *J Biomol Nmr* **6**, 277-293, doi:Doi 10.1007/Bf00197809 (1995).

Table S1

Table S1. Accession number of sequences deposited in DDBJ

Plot name[a]	Leaf blade	Leaf sheath
AMBI-AT	AB717425-AB717570	AB718353-AB718516
AMBI-ET	AB717139-AB717300	AB717999-AB718172
AMBI-LN	AB717301-AB717424	AB718173-AB718352
FACE-AT	AB717848-AB717998	AB718876-AB719043
FACE-ET	AB717571-AB717721	AB718517-AB718694
FACE-LN	AB717722-AB717847	AB718695-AB718875

[a]Plot name stands for the combination of following environmental factors; AMBI, ambient CO_2; FACE, free air CO_2 enrichment; AT, ambient surface water-soil temperature; ET, elevated surface water-soil temperature; LN, low nitrogen fertilization. See the main text for the detailed description for abbreviations of environmental factors.

Table S2

Table S2. Major genera for leaf blade- and leaf sheath-associated bacteria in rice plants cultivated under different environmental conditions

	Relative abundance in a clone library (%)[a]											
Tissues	Leaf blade						Leaf sheath					
Atmospheric conditions	Ambient[b]			FACE[c]			Ambient			FACE		
Temperature	NT[d]	ET[e]	LN[f]	NT	ET	LN	NT	ET	LN	NT	ET	LN
Alphaproteobacteria	30.8	19.1*	24.2	33.1	31.1	15.9**	29.9	29.9	32.8	29.8	29.2	33.7
Methylobacterium	4.8	4.3	0.8	6.0	4.6	1.6	2.4	6.9	14.4**	8.3*	9.0*	11.6**
Phyllobacterium	5.5	-**	-**	-**	-**	-**	-	-	-	-	-	-
Rhizobium	14.4	4.4**	17.7	15.9	17.2	9.5	15.9	15.5	13.9	12.5	7.9*	16.6
Sphingomonas	4.1	9.9	4.0	8.6	7.9	4.8	3.7	1.1	-*	6.0	3.9	1.1
Betaproteobacteria	3.4	5.6	-	8.6	7.9	1.6	4.9	9.2	5.0	7.7	10.7*	3.3
Herbaspirillum	2.1	3.1	-	4.6	3.3	-	1.8	4.0	1.1	4.8	2.2	1.1
Acidovorax	1.4	2.5	-	3.3	4.6	1.6	3.0	4.0	3.3	1.2	6.7	1.7
Gammaproteobacteria	37.7	46.3	32.3	45.0	37.1	58.7**	14.0	13.8	22.2	11.3	15.2	18.2
Pantoea	30.8	43.8*	25.0	39.7	33.1	52.4**	8.5	8.6	7.8	8.3	11.8	9.9
Aquicella	0.7	1.2	-	-	1.3	0.8	-	1.1	1.1	1.2	1.7	2.8*
Planctomycetes	1.4	3.7	-	0.7	1.4	0.8	30.5	27.6	20.0*	25.6	22.5	22.6
Planctomyces	-	1.2	-	-	-	-	9.1	10.3	6.1	8.9	7.9	8.8
Pirellula	-	1.2	-	-	0.7	0.8	9.8	10.9	9.4	9.5	7.9	10.5
Rhodopirellula	-	-	-	-	-	-	6.1	2.3	-**	3.6	3.9	2.8
Bacilli	11.6	15.5	27.4**	6.0	8.6	13.5	1.8	0.6	1.7	3.0	3.3	1.7
Paenibacillus	2.7	1.9	1.6	2.6	2.0	0.8	-	-	1.1	0.6	0.6	-
Bacillus	1.4	3.7	2.4	0.7	1.3	0.8	0.6	-	-	-	0.6	-
Staphylococcus	2.7	1.2	3.2	0.7	2.0	2.4	0.6	-		1.2	1.7	0.6
Exiguobacterium	4.8	4.3	18.5**	1.3	2.6	5.6	0.6	0.6	-	1.2	0.6	0.6
Clostridia	5.5	3.7	8.9	2.0	5.3	1.6	2.4	0.6	1.1	2.4	2.8	2.2
Clostridium XlVa	1.4	0.6	4.0	-	2.6	-	-	-	-	-	0.6	-
Sporobacter	1.4	0.6	0.8	-	0.7	-	-	-	-		-	-
Clostridium III	-	-	-	0.7	0.7	-	0.6	-	-	0.6	-	-
Clostridium sensu stricto	2.7	1.9	0.8	0.7	2.0	1.6	-	-	-	0.6	1.1	2.2
Clostridium XI	-	-	0.8	-	-	-	-	-	-	-	-	-

[a]* and ** indicate statistical significance at the 1 and 5% levels ($P < 0.01$ and $P < 0.05$), respectively, calculated with the Library Compare of RDP II, between NT sample under ambient atmosphere and other samples.

[b]Rice plants grown under ambient atmosphere condition.

[c]Rice plants grown under the free-air CO_2 enrichment condition.

[d]Rice plants grown in normal surface water-soil temperature with standard nitrogen fertilization.

[e]Rice plants grown in elevated surface water-soil temperature (2 °C above NT) with standard nitrogen fertilization.

[f]Rice plants grown in normal surface water-soil temerature with no nitrogen fertilization.

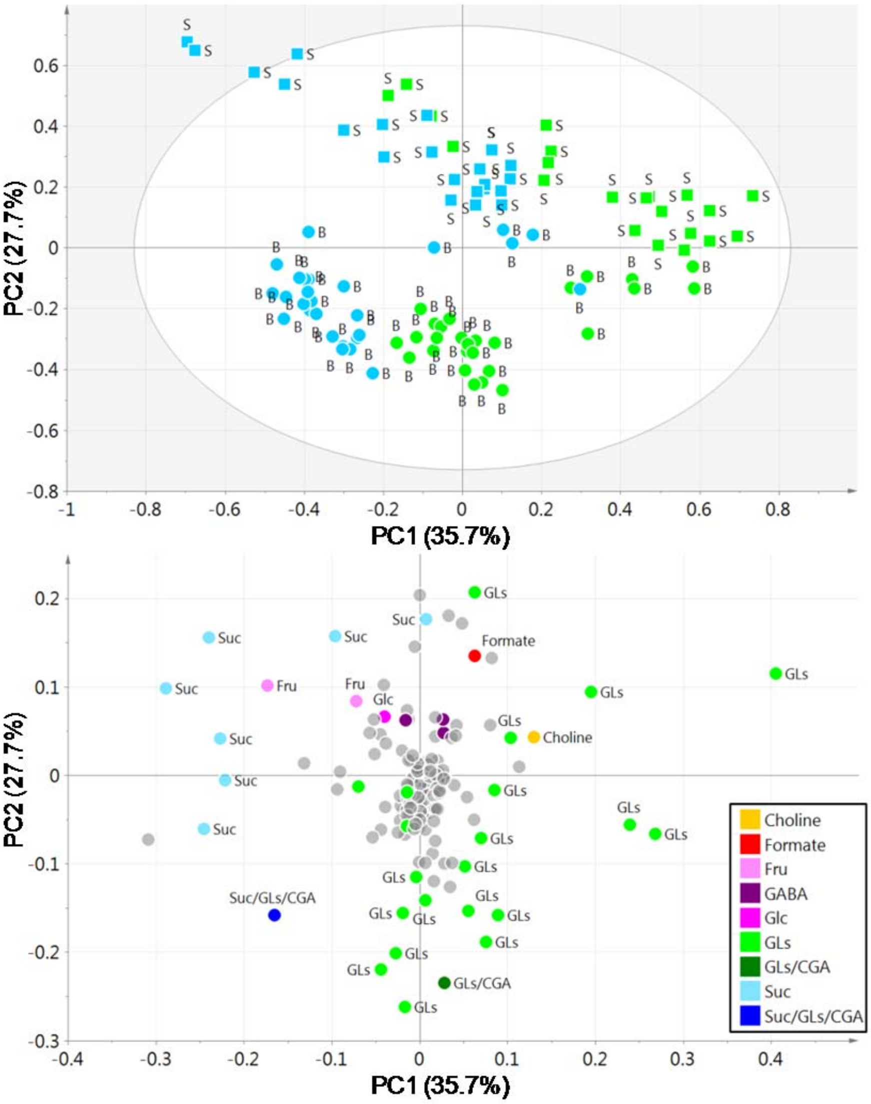

Fig. S-1. Principal component analysis of NMR spectra for rice shoot metabolites. Panel A; PCA score plot, leaf blade (circle) and sheath (rectangle) grown under ambient (green) and FACE (blue) conditions. Panel B; loading plot. The variables corresponding to choline, formate, Fru (fructose), GABA (4-aminobutyrate), Glc (glucose), GLs (glycerolipids), the bucket containing GLs and chlorogenate (CGA), Suc (sucrose), the bucket containing Suc, GLs, and CGA, were colored as shown on the right.
